# Detection and characterization of multidrug resistant *Escherichia coli* carrying virulence gene isolated from broilers in Bangladesh

**DOI:** 10.1002/vms3.70032

**Published:** 2024-09-18

**Authors:** Md. Sirazul Islam, Chandan Nath, F. M. Yasir Hasib, Tahia Ahmed Logno, Md. Helal Uddin, Mohammad Mahmudul Hassan, Sharmin Chowdhury

**Affiliations:** ^1^ Department of Pathology and Parasitology Faculty of Veterinary Medicine Chattogram Veterinary and Animal Sciences University Chattogram Bangladesh; ^2^ Department of Microbiology and Veterinary Public Health Faculty of Veterinary Medicine Chattogram Veterinary and Animal Sciences University Chattogram Bangladesh; ^3^ Department of Medicine and Surgery Faculty of Veterinary Medicine Chattogram Veterinary and Animal Sciences University Chattogram Bangladesh; ^4^ Queensland Alliance for One Health Sciences School of Veterinary Science The University of Queensland Gatton Queensland Australia; ^5^ Department of Physiology Biochemistry and Pharmacology Chattogram Veterinary and Animal Sciences University Chattogram Bangladesh; ^6^ Melbourne Veterinary School Faculty of Science University of Melbourne Parkville Australia

**Keywords:** AMR, ARGs, chicken, public health, VAGs

## Abstract

**Background:**

The emergence and dissemination of multidrug resistant (MDR) bacteria pose a severe threat to public health by limiting clinical treatment and prophylactic options.

**Objectives:**

This study investigates the prevalence of *Escherichia coli* in broilers, their phenotypic antimicrobial resistance (AMR) profiles and the presence of virulence‐associated genes (VAGs) and antimicrobial resistance genes (ARGs) using polymerase chain reaction (PCR).

**Materials and methods:**

A total of 216 pooled cloacal samples were collected from 1080 broilers across six districts of Bangladesh. Each pooled sample comprised randomly selected cloacal swabs from five birds per farm. *E. coli* isolates were identified using standard bacteriological approach, followed by biochemical assays and PCR. Antimicrobial susceptibility was assessed using the Kirby–Bauer disc diffusion method, and the presence of ARGs and VAGs was determined via PCR. Five selected isolates were partially sequenced for five VAGs using Sanger sequencing.

**Results:**

A total of 177 *E. coli* isolates (81.94%, 95% confidence interval: 76.24%–86.53%) were identified. The isolates showed the highest resistance to ampicillin (93.79%), followed by tetracycline (91.53%), erythromycin (89.27%) and ciprofloxacin (87%). Conversely, ceftriaxone (80.79%) showed highest susceptibility, followed by gentamicin (37.29%) and neomycin (31.07%). All isolates were MDR, with a multiple antibiotic resistance indexes were <0.3. A significant percentage (16.38%) of *E. coli* isolates were MDR to five antimicrobial classes and harboured *bla*_TEM_, *sul*1, *ere* (A), *tet*A, *tet*B and *tet*C genes. The highest prevalent ARGs were *bla*_TEM_ (88.14%) followed by *ere* (A) (83.62%) and *sul* 1 (72.32%). The prevalence of VAGs was *ast*A (56.50%), *iuc*D (31.07%), *iss* (21.47%), *irp*2 (15.82%) and *cva/cvi* (3.39%), respectively.

**Conclusions:**

This study highlights the presence of ARGs contributing to the development of MDR in *E. coli* carrying VAGs in broilers. Effective monitoring and surveillance of antimicrobial usage in poultry production systems are urgently required to prevent emergence and dissemination of AMR.

## Graphical abstract

This study investigated *E. coli* prevalence, AMR and VAGs isolated from broilers in Bangladesh. The prevalence of *E. coli* was 81.94%. The isolates exhibited the highest resistance to ampicillin (94%) and the lowest resistance to ceftriaxone (19%). All the isolates were MDR, and the correlation coefficient showed a significant correlation (*r* = 0.8) between the *aac (3)*‐IV gene and gentamicin, the *ere* (A) gene and erythromycin. The most prevalent ARG was *bla*_TEM_ (88.14%), and VAG was *ast*A (56.5%).

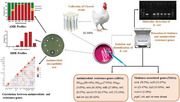

## 1 INTRODUCTION

*Escherichia coli*, which has been considered a harmless and versatile microbe, can lead to various intestinal and extra‐intestinal diseases, including haemorrhagic diarrhoea, urinary tract infections, meningitis and pneumonia, in humans and animals by acquiring different virulence traits (Hammerum & Heuer, 2009; Kunert Filho et al., 2015; Parvin et al., 2020). Commonly used for treating and preventing diseases in poultry, antimicrobials were used for growth promotion in poultry production, leading to antimicrobial misuse (Hassan et al., 2021). Bacteria have acquired drug resistance genes through horizontal gene transfer or gene mutation mechanisms (Darby et al., 2023). The poor selection and irrational use of antimicrobials are the leading causes of the emergence and dissemination of antimicrobial resistant bacteria, which can potentially be transmitted to humans through the food chain (Hasib et al., 2024; Parvin et al., 2020). According to the World Health Organization (WHO), millions of patients are expected to die in the coming decades due to antimicrobial resistance (AMR), as antimicrobials fail to treat clinical cases of infections (WHO, 2023).

Bangladesh, a major poultry producer, according to the Department of Livestock Service Economy 2019–2020, relies on the poultry industry to drive economic growth in rural communities. In this context, approximately 3563.18 lakhs of poultry were generated from a total livestock production of 4122.44 lakhs (DLS, 2020). The extent of antimicrobial usage (AMU) in poultry and the annual sales data of antimicrobials in Bangladesh remain unknown (Imam et al., 2020). Farmers can easily acquire antimicrobials from drug sellers without needing a prescription from a registered veterinarian (Ahmed et al., 2020). Despite recent stringent regulations on antimicrobial sales, unregulated use of antimicrobials is widespread in poultry production in Bangladesh (Hassan et al., 2021).

Multidrug resistance has been increased all over the world that is considered a public health threat. Several recent investigations reported the emergence of multidrug resistant (MDR) bacterial pathogens from different origins that increase the necessity of the proper use of antibiotics. Besides, the routine application of the antimicrobial susceptibility testing (AST) to detect the antibiotic of choice as well as the screening of the emerging MDR strains (Algammal et al., 2019, 2022, 2023; Elbehiry et al., 2022; Shafiq et al., 2022).

Pathogenicity is expedited by virulence factors like adhesion, iron acquisition, haemolysin, aerobactin's and serum resistance translated by virulence‐associated genes (VAGs) found in plasmids or chromosomal areas of pathogenicity islands (PAIs) (Subedi et al., 2018). These virulence factors permeate host tissues, produce toxins, evade host defences and ultimately cause local inflammation in the host, among other pathways that lead to infection (El‐Baz et al., 2022). Isolates with at least five virulence genes are classified as avian pathogenic *E. coli* (APEC), and five genes (*iutA, iss, ompT, iroN* and *hlyF*) mainly were associated with pathogenesis (De Carli et al., 2015).

*E. coli* also causes colibacillosis, resulting in considerable disease in the poultry industry worldwide (Kika et al., 2023). The impacts include production losses, increased mortality and higher production costs due to systemic infections in the poultry gut, characterized by acute fatal septicemia or sub‐acute fibrinous pericarditis, air sacculitis, salpingitis and peritonitis (Ibrahim et al., 2019). Moreover, the presence of antimicrobial resistance genes (ARGs) and VAGs in the farm makes it more vulnerable to contaminating the surrounding environment and adds selection pressure on the environmental pathogens. Maintaining effective biosecurity on broiler farms appears to be impractical with current farming practices in Bangladesh, which would exacerbate the situation in terms of potential zoonotic pathogen transmission to humans and other animals.

AMR is a burning issue, and there is a lack of stringent strategies to control AMU in poultry production in Bangladesh. Given the importance of AMR and VAGs in *E. coli*, this study was undertaken to estimate the prevalence of *E. coli* from cloacal samples of broiler chickens. Subsequently, the phenotypic AMR profile was examined, followed by molecular identification of ARGs and VAGs to establish a baseline of AMR patterns and VAGs found in *E. coli* isolated from broiler chickens in Bangladesh.

## 2 MATERIALS AND METHODS

### 2.1 Sampling

**FIGURE 1:**
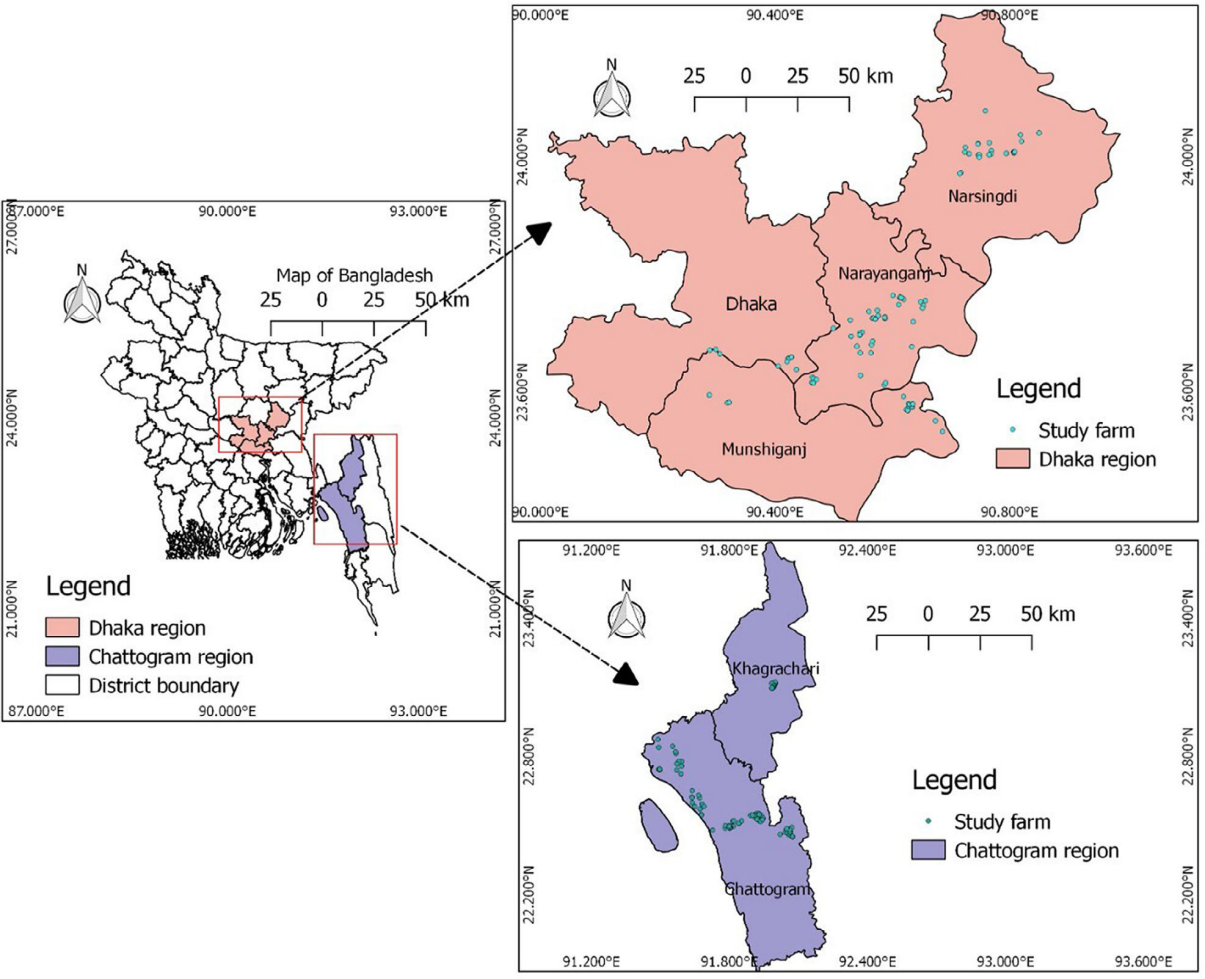
A map of Bangladesh displaying the geographical location of broiler farms included in the study.

This cross‐sectional study was conducted between June 2019 and March 2020 in six districts of Bangladesh: Chattogram, Dhaka, Narsingdi, Narayanganj, Munshigonj and Khagrachari. The first five districts are plain land, and the last one is a hilly area. The districts were selected based on their poultry population density (Figure 1). Broiler farms were chosen randomly from the list provided by the government district livestock office. The following formula was used for the sample size calculation:

$$\mathrm{Sample}\;\mathrm{size},\;n=N\times \frac{\frac{Z^{2}\times p\times \left(1-p\right)}{e^{2}}}{\left[N-1+\frac{Z^{2}\times p\times \left(1-p\right)}{e^{2}}\right]}$$

where *n* is the sample size, *N* is the population size, critical value (95% level of significance) *Z* is the 1.96 s, margin of error *e* is the 0.05 and sample proportion *p* is the 0.5.

Later, one pooled cloacal swab sample was collected for each of the representative farms from five randomly selected birds from the broiler flocks. Swabs were taken aseptically using commercial swabs (Model: PW005 Himedia) and transferred into the falcon tube containing 5 mL buffered peptone water (BPW) (Oxoid Ltd.). All samples were stored at 4°C and transferred carefully for laboratory processing (not more than 24 h) and subsequent isolation.

### 2.2 Isolation and identification of *E. coli*

Isolation and identification were performed following standard bacteriological methods (Dutta et al., 2020). Briefly, all samples were pre‐enriched overnight in BPW (Oxoid Ltd.). A loopful broth (∼10 µL) was inoculated onto MacConkey agar and eosin‐methylene blue (EMB) agar (Oxoid Ltd.) and incubated overnight at 37°C. The purified colonies were sub‐cultured onto blood agar with 5% bovine blood (Oxoid Ltd.) and incubated overnight at 37°C. Colonies were further confirmed by Gram stain properties and several biochemical tests (catalase, oxidase, lactose fermentation, Voges‐Proskauer, indole, methyl‐red and H_2_S production). A single‐isolated colony was preserved at −80°C in equal ratio (1:1) of brain heart infusion broth (Oxoid Ltd.) and 50% buffered glycerine.

### 2.3 Molecular confirmation of *E. coli*

The genomic DNA was extracted following the conventional crude boiling method (Malorny et al., 2003). A polymerase chain reaction (PCR) assay was conducted for the final confirmation of the suspected isolates using a genus‐specific universal primer targeting the *16S rRNA* gene: (F) 5′‐GACCTCGGTTTAGTTCACAGA‐3′ and (R) 5′–CACACGCTGACGCTGACCA‐3′, maintaining the initial denaturation at 95°C for 5 min and final extension at 72°C for 7 min with the 35 cycles of denaturation at 94°C for 1 min, annealing at 58°C for 1 min and extension at 72°C for 1 min (Schippa et al., 2010). The PCR products were visualized on a gel documentation system (UVP UVsolo touch‐Analytik Jena AG, Thermo Fisher Scientific) after electrophoresis with a 1.5% agarose gel (MP Biomedicals).

### 2.4 Phenotypic antimicrobial susceptibility test (AST)

All *E. coli* isolates were tested for AST following the Kirby–Bauer disc diffusion method (Bauer et al., 1966). Eight antimicrobials of seven different classes – cephalosporins: ceftriaxone (CRO, 30 µg), penicillins: ampicillin (AMP, 10 µg), fluoroquinolones: ciprofloxacin (CIP, 5 µg), aminoglycosides: gentamicin (CN, 10 µg) and neomycin (*N*, 30 µg), macrolides: erythromycin (*E*, 15 µg), sulphonamides: sulfamethoxazole‐trimethoprim (SXT, 23.75 + 1.25 µg) and tetracycline: tetracycline (TE, 30 µg) (Oxoid Ltd.) were used. The zone diameter was measured using slide calipers and interpreted in accordance with CLSI criteria (CLSI, 2020). *E. coli* ATCC25922 was used as a quality control organism. *E. coli* isolates resistant to at least three antimicrobial classes were classified as MDR (Magiorakos et al., 2012). Additionally, multiple antibiotic resistance (MAR) indexes were calculated using the following formula: a/b (a: a number of antimicrobial agents to which the isolates are resistant/b: total number of tested antimicrobial agents), as previously described by Algamma et al. (2022).

### 2.5 Detection of AMR genes (ARGs)

Although phenotypically eight antimicrobials were tested in this study, the following ARGs were screened from the isolates – aminoglycoside resistant [*aac*(3*)‐*IV], sulphonamide resistant (*sul*1), penicillin resistant (*bla*_SHV_, *bla*_CMY_, *bla*_TEM_), erythromycin resistant [*ere* (A)] and TE resistant (*tet*A*, tet*B*, tet*C) by PCR. The oligonucleotide primers sequence and cyclic conditions used for each gene are shown in Table 1.

**TABLE 1:** The oligonucleotide primer sequences for detection of antimicrobial resistance genes of *Escherichia coli*.

Gene	Primer sequence (5′–3′)	Size (bp)	Cyclic conditions (35 cycles)			References
			Denatur.	Annealing	Extension	
*aac(3)‐IV* (gentamicin resistance)	F: CTTCAGGATGGCAAGTTGGT	286	94°C for 1 min	55°C for 90 s	72°C for 1 min	Momtaz et al. (2012)
	R: TCATCTCGTTCTCCGCTCAT					
*sul1* (sulphonamide resistance)	F: TTCGGCATTCTGAATCTCAC	822	94°C for 1 min	47°C for 90 s	72°C for 1 min	
	R: ATGATCTAACCCTCGGTCTC					
*bla*_SHV_ (penicillin resistance)	F: TCGCCTGTGTATTATCTCCC	768	94°C for 1 min	52°C for 90 s	72°C for 1 min	
	R: CGCAGATAAATCACCACAATG					
*bla*_CMY_ (penicillin resistance)	F: TGGCCAGAACTGACAGGCAAA	462	94°C for 1 min	47°C for 90 s	72°C for 1 min	
	R: TTTCTCCTGAACGTGGCTGGC					
*bla*_TEM_ (penicillin resistance)	F: GCGGAACCCCTATTTG	964^*^	94°C for 3 min	50°C for 60 s	72°C for 1 min	Hasman et al. (2005)
	R: TCTAAAGTATATATGAGTAAA CTTGGTCTGAC					
*ere(A)* (erythromycin resistance)	F: GCCGGTGCTCATGAACTTGAG	419	94°C for 1 min	52°C for 90 s	72°C for 1 min	Momtaz et al. (2012)
	R: CGACTCTATTCGATCAGAGGC					
*tetA* (tetracycline resistance)	F: GGTTCACTCGAACGACGTCA	577	94°C for 1 min	57°C for 90 s	72°C for 1 min	
	R: CTGTCCGACAAGTTGCATGA					
*tetB* (tetracycline resistance)	F: CCTCAGCTTCTCAACGCGTG	634	94°C for 1 min	56°C for 90 s	72°C for 1 min	
	R: GCACCTTGCTGATGACTCTT					
*tetC* (tetracycline resistance)	F: AAC AAT GCG CTC ATC GT	1138	95°C for 30 s	58°C for 60 s	72°C for 1 min	Kim et al. (2013)
	R: GGA GGC AGA CAA GGT AT					

* 25 cycles.

### 2.6 Detection of virulence‐associated genes (VAGs)

All confirmed *E. coli* isolates were further investigated for targeted VAGs by multiplex PCR (Ewers et al., 2007). The primer sequences used for the PCR confirmation are summarized in Table 2. The reaction volume mixture was 25 µL containing 12.5 µL Taq 2X master mix (New England Biolabs Inc.), 1 µL forward and reverse primer (20 pmol/µL), 2 µL template DNA (average 3.35 ng/µL DNA) and 8.5 µL nuclease‐free water. PCR was run on a thermocycler (Applied Biosystem, 2720 thermal cycler) following the cycling conditions: the initial denaturation at 94°C for 3 min and final extension at 72°C for 10 min with the 35 cycles of denaturation at 94°C for 30 s, annealing at 58°C for 30 s and extension at 68°C for 3 min.

**TABLE 2:** Oligonucleotide primer sequences for detection of target virulence genes of *Escherichia coli* isolates.

Gene	Descriptions	Primer sequence (5′–3′)	Size (bp)	References
*ast*A	EAST1 (heat‐stable cytotoxin associated with enteroaggregative *E. coli*)	F: TGCCATCAACACAGTATATCC	116	Ewers et al. (2007)
		R: TCAGGTCGCGAGTGACGGC		
*iss*	Increased serum survival	F: ATCACATAGGATTCTGCCG	309	
		R: CAGCGGAGTATAGATGCCA		
*irp*2	Iron‐repressible protein (yersiniabactin synthesis)	F: AAGGATTCGCTGTTACCGGAC	413	
		R: AACTCCTGATACAGGTGGC		
*pap*C	Pilus associated with pyelonephritis	F: TGATATCACGCAGTCAGTAGC	501	
		R: CCGGCCATATTCACATAA		
*iuc*D	Aerobactin synthesis	F:ACAAAAAGTTCTATCGCTTCC	714	
		R: CCTGATCCAGATGATGCTC		
*tsh*	Temperature‐sensitive haemagglutinin	F:ACTATTCTCTGCAGGAAGTC	824	
		R: CTTCCGATGTTCTGAACGT		
*vat*	Vacuolating autotransporter toxin	F:TCCTGGGACATAATGGTCAG	981	
		R:GTGTCAGAACGGAATTGT		
*cva/cvi*	Structural genes of colicin V operon (microcin ColV)	F:TGGTAGAATGTGCCAGAGCAAG	1181	
		R: GAGCTGTTTGTAGCGAAGCC		

### 2.7 Sequencing of virulence‐associated genes (VAGs)

Five VAGs (*ast*A*, iss, irp*2*, iuc*D and *cvi/cva*C) from five *E. coli* isolates were randomly selected for partial sequencing. The PCR products were purified using a DNA purification kit (Favorgen Biotech Corp.) and sequenced both forward and reverse strands by sanger method using a commercial service (Macrogen Inc.). The raw forward and reverse sequence read data were manually cleaned up with a chromatogram and assembled by CAP3 sequence alignment programme (Huang & Madan, 1999) to make consensus sequence. The consensus sequences were then deposited in the NCBI GenBank database for accession numbers (Accession numbers: MT928164‐MT928166, MT982360‐MT982361).

### 2.8 Statistical analysis

Data retrieved from the samples were inserted in the Microsoft Office Excel 2016 Excel sheet. The frequency (*n*), prevalence (%) and 95% confidence intervals (CIs) were calculated using the modified Wald method in the GraphPad software QuickCalcs (https://www.graphpad.com/quickcalcs/). The map and the farm's location were created using QGIS 3.12.0. The correlation coefficient between phenotypic and genotypic resistance was calculated using R software (version 4.4.1; https://www.r‐project.org/).

## 3 RESULTS

AMR and VAGs in *E. coli* are major public health concerns worldwide, particularly in countries like Bangladesh, where poultry farming is popular. This study sought to assess the AMR patterns and VAG profiles of *E. coli* isolated from broiler cloacal samples in Bangladesh. This study hypothesized that *E. coli* isolated from broiler would be MDR and have several VAGs, increasing their harmful potential. Thus, we collected cloacal swabs from broilers on farms in various regions of Bangladesh, isolated *E. coli* and assessed antibiotic susceptibility and VAGs using PCR.

### 3.1 Phenotypic characteristics and prevalence of recovered *E. coli* isolates

**FIGURE 2:**
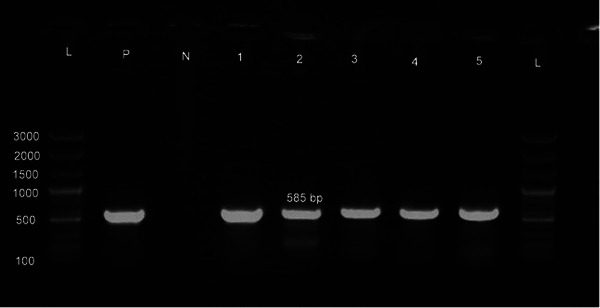
Result of polymerase chain reaction (PCR) assay for the *16s rRNA* gene of *Escherichia coli* isolates tested; Lane L: 1 kb plus DNA ladder; Lane P: positive control; Lane N: negative control; Lane 1–5: *16S rRNA* gene‐size (585 bp) amplicon.

All *E. coli* isolates recovered in this study were Gram‐negative, motile and rod shaped bacilli. The colonies were large, pink, lactose fermentative on MacConkey agar, showed metallic sheen on EMB agar and were hemolytic on blood agar. Additionally, all the isolates tested positive for catalase, lactose fermentation, indole and methyl red tests. Conversely, they tested negative for oxidase, H_2_S production and Voges–Proskauer test. A total of 216 pooled broiler cloacal samples were investigated. Among them, 177 of 216 samples (81.94%; 95% CI 76.24–86.53) were confirmed by PCR to be colonized with *E. coli* (Figure 2).

### 3.2 AMR profiles

**FIGURE 3:**
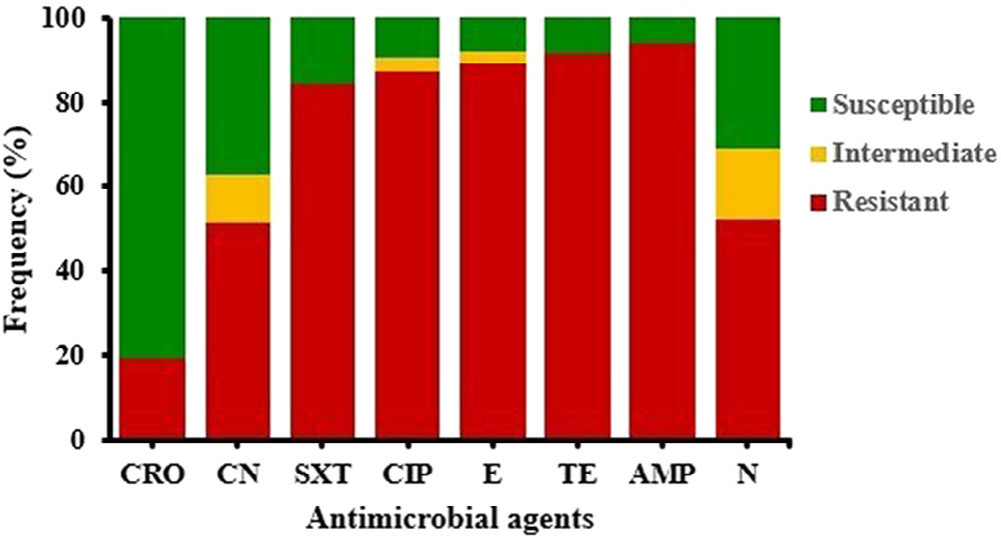
Antimicrobial resistance profiles of *Escherichia coli* isolates. AMP, ampicillin; CIP, ciprofloxacin; CN, gentamicin; CRO, ceftriaxone; E, erythromycin; *N*, neomycin; SXT, sulfamethoxazole/trimethoprim; TE, tetracycline.

The AMR profiles of *E. coli* isolates are illustrated in Figure 3. The isolates showed the significant resistance to ampicillin (93.79%), followed by tetracycline (91.53%) and erythromycin (89.27%). On the other hand, they showed the highest susceptibility to ceftriaxone (80.79%), followed by gentamicin (37.29%) and neomycin (31.07%). Besides, the isolates showed remarkable resistance to sulfamethoxazole/trimethoprim (84.18%) and ciprofloxacin (87%), respectively.

### 3.3 Phenotypic MDR profiles and distribution of ARGs

**FIGURE 4:**
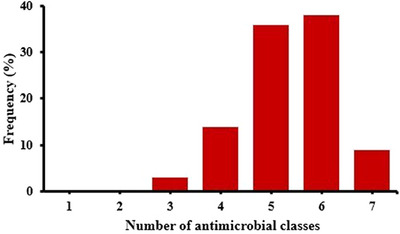
Multidrug resistant (MDR) patterns of *Escherichia coli* isolated from broiler chicken farms.

All the isolates in this study were MDR (resistance against ≥3 antimicrobial classes), as illustrated in Figure 4. This study showed that 16.38% (29/177) of isolates were MDR to five antimicrobial classes carried *bla*_TEM_ *_,_ sul* 1, *ere* (A), *tet* A, *tet* B and *tet* C genes. Meanwhile, 15.82% (28/177) recovered isolates were MDR to 6 classes and possessed *bla*_TEM_ *_,_ sul*1, *ere* (A), *tet* A, *tet* B, *tet* C and *aac* (3)‐IV genes. Besides, 3.95% (7/177) and 3.39% (6/177) of isolates were MDR to 7 classes and carried *bla*_TEM_ *_,_ bla*_SHV_, *bla*_CMY_, *sul*1, *ere* (A), *tet* A, *tet* B and *aac*(3)‐IV genes, as illustrated in Table 3.

**TABLE 3:** The occurrence of multidrug resistant (MDR) among the recovered *Escherichia coli* isolates.

No. of strains	%	Resistance type	Phenotypic multidrug resistance	Antibiotic resistance genes	MAR index
29	16.38	MDR	Five classes AMP, SXT, E, CIP and TE	*bla*_TEM_ *_,_ sul1*, *ere(A)*, *tetA*, *tetB* and *tetC*	0.63
28	15.82	MDR	Six classes AMP, SXT, E, CIP, TE, CN and N	*bla*_TEM_ *_,_ sul1*, *ere(A)*, *tetA*, *tetB*, *tetC* and *aac(3)‐IV*	0.88
18	10.17	MDR	Six classes AMP, SXT, CIP, TE, E and N	*bla*_TEM_ *_,_ sul1*, *ere(A)*, *tetA*, *tetB* and *tetC*	0.75
11	6.21	MDR	Six classes AMP, SXT, E, CIP, TE and CN	*bla*_TEM_ *_,_ sul1*, *ere(A)*, *tetA*, *tetB*, *tetC* and *aac(3)‐IV*	0.75
7	3.95	MDR	Seven classes CRO, AMP, SXT, E, CIP, TE and CN	*bla*_TEM_ *_,_ bla*_SHV_, *bla*_CMY_, *sul1*, *ere(A)*, *tetA*, *tetB* and *aac(3)‐IV*	0.88
6	3.39	MDR	Seven classes CRO, AMP, SXT, E, CIP, TE, CN and N	*bla*_TEM_ *_,_ bla*_SHV_, *bla*_CMY_, *sul1*, *ere(A)*, *tetA*, *tetB* and *aac(3)‐IV*	1
5	2.82	MDR	Five classes AMP, SXT, CIP, TE, CN and N	*bla*_TEM_ *_,_ sul1*, *tetA*, *tetB*, *tetC* and *aac(3)‐IV*	0.75
4	2.26	MDR	Four classes AMP, E, SXT and CIP	*bla_TEM,_ sul1*, *ere(A)*	0.75
4	2.26	MDR	Five classes AMP, SXT, E, TE, CN and N	*bla*_TEM_ *_,_ sul1*, *ere(A)*, *tetA* and *aac(3)‐IV*	0.75
4	2.26	MDR	Four classes SXT, E, TE and CIP	*sul1*, *ere(A)*, *tetA*, *tetB* and *tetC*	0.5
3	1.69	MDR	Six classes CRO, AMP, SXT, CIP, TE and E	*bla_TEM,_ bla_SHV_*, *bla_CMY_*, *sul1*, *ere(A)*, *tetA* and *tetB*	0.75
3	1.69	MDR	Five classes AMP, E, TE, CIP, CN and N	*bla*_TEM_ *_,_ ere(A)*, *tetA*, *tetB* and *aac(3)‐IV*	0.75
3	1.69	MDR	Five classes AMP, SXT, E, TE and N	*bla_TEM,_ sul1*, *ere(A)*, *tetA, tetB* and *aac(3)‐IV*	0.63
3	1.69	MDR	Five classes AMP, SXT, CIP, TE and N	*bla_TEM,_ sul1*, *tetA*, *tetB* and *tetC*	0.63
3	1.69	MDR	Four classes SXT, E, TE, CN and N	*sul1*, *ere(A)*, *tetA* and *aac(3)‐IV*	0.63
3	1.69	MDR	Four classes AMP, SXT, CIP and N	*bla_TEM_* and *sul1*	0.5
3	1.69	MDR	Four classes E, TE, CIP and N	*ere(A)*, *tetA* and *aac(3)‐IV*	0.5
3	1.69	MDR	Seven classes CRO, AMP, SXT, E, CIP, TE and N	*bla*_TEM_ *_,_ bla*_CMY_, *sul1*, *ere(A)*, *tetA*, *tetB* and *tetC*	0.88
2	1.13	MDR	Six classes CRO, AMP, SXT, E, TE, CN and N	*bla*_TEM_ *_,_ bla*_CMY_, *sul1*, *ere(A)*, *tetA* and *aac(3)‐IV*	0.88
2	1.13	MDR	Six classes CRO, AMP, E, CIP, TE and CN	*bla*_TEM_ *_,_ bla*_CMY_, *ere(A)*, *tetA* and *aac(3)‐IV*	0.75
2	1.13	MDR	Six classes CRO, AMP, SXT, E, CIP, CN and N	*bla*_CMY_, *sul1*, *ere(A)* and *aac(3)‐IV*	0.88
2	1.13	MDR	Five classes CRO, AMP, E, CIP and TE	*bla*_TEM_ *_,_ ere(A)*, *tetA* and *aac(3)‐IV*	0.63
2	1.13	MDR	Five classes SXT, E, CIP, TE, CN and N	*sul1*, *ere(A)*, *tetA*, *tetB* and *aac(3)‐IV*	0.75
2	1.13	MDR	Five classes AMP, SXT, E, TE and CN	*bla*_TEM_ *_,_ sul1*, *ere(A)*, *tetA* and *aac(3)‐IV*	0.63
2	1.13	MDR	Five classes AMP, E, CIP, TE and N	*bla*_TEM_ *_,_ ere(A)*, *tetA* and *tetB*	0.63
2	1.13	MDR	Five classes AMP, E, SXT, CIP and N	*bla*_TEM_ *_,_ sul1* and *ere(A)*	0.63
2	1.13	MDR	Five classes CRO, AMP, E, TE and CN	*bla*_TEM_ *_,_ ere(A)* and *tetA*	0.63
2	1.13	MDR	Five classes CRO, AMP, E, TE and N	*bla*_TEM_ *_,_ bla*_SHV_, *bla*_CMY_, *ere(A)* and *tetA*	0.63
2	1.13	MDR	Four classes AMP, E, TE and CIP	*bla*_TEM,_ *ere(A)*, *tetA*, *tetB* and *tetC*	0.5
2	1.13	MDR	Four classes AMP, SXT, TE and CIP	*bla*_TEM_ and *tetA*	0.5
2	1.13	MDR	Four classes AMP, E, TE and CN	*bla*_TEM_ *_,_ ere(A)*, *tetA*, *tetB* and *aac(3)‐IV*	0.5
2	1.13	MDR	Four classes AMP, SXT, CIP, CN and N	*bla*_TEM_ and *sul1*	0.63
2	1.13	MDR	Three classes AMP, E and TE	*bla*_TEM_ *_,_ ere(A)* and *tetA*	0.38
1	0.56	MDR	Three classes AMP, CIP and E	*bla*_TEM_ and *ere(A)*	0.38
1	0.56	MDR	Three classes E, TE, CN and N	*ere(A)*, *tetA*, *tetB* and *aac(3)‐IV*	0.5
1	0.56	MDR	Three classes AMP, TE and CN	*bla*_TEM_ *_,_ tetA* and *aac(3)‐IV*	0.38
1	0.56	MDR	Six classes CRO, AMP, SXT, CIP, TE and CN	*bla*_TEM_ *_,_ bla_SHV_*, *sul1*, *ere(A)*, *tetA*, *tetB* and *aac(3)‐IV*	0.75
1	0.56	MDR	Five classes AMP, SXT, E, CIP, CN and N	*bla*_TEM_ *_,_ sul1* and *aac(3)‐IV*	0.63
1	0.56	MDR	Five classes AMP, E, TE, CIP and CN	*bla*_TEM_ and *ere(A)*	0.63
1	0.56	MDR	Three classes AMP, CIP and TE	*bla*_TEM_	0.38

**FIGURE 5:**
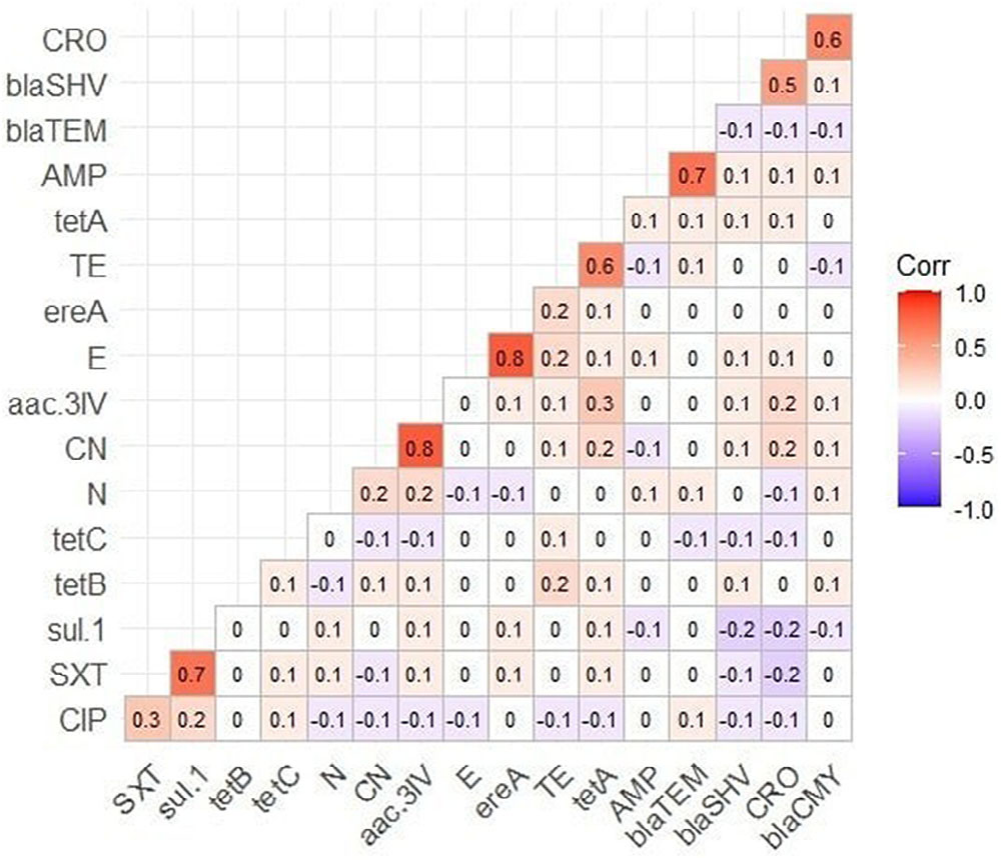
The heatmap represents the correlation between antimicrobial agents and antimicrobial resistance genes (ARGs).

Moreover, the MAR index value in this study was >0.3, which indicated that recovered *E. coli* strains originated from high risk contamination. Furthermore, the correlation coefficient among phenotypic AMR and ARGs were estimated and revealed positive correlation between *sul*1 gene and SXT (*r* = 0.7), *aac*(3)‐IV gene and CN (*r* = 0.8), *ere* (A) gene and E (*r* = 0.8), *tet* A gene and TE (*r* = 0.6), *bla*_TEM_ gene and AMP (*r* = 0.7), *bla*_SHV_ gene and CRO (*r* = 0.5) and *bla*_CMY_ gene and CRO (*r* = 0.6), as illustrated in Figure 5.

**FIGURE 6:**
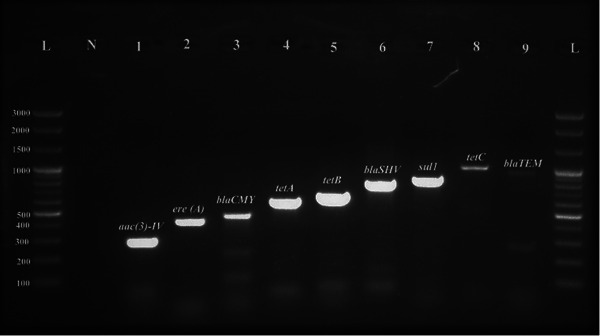
Result of polymerase chain reaction (PCR) assay for the antimicrobial resistance genes of *Escherichia coli* isolates tested; Lane L: 1 kb plus DNA ladder; Lane N: negative control; Lane 1–9: samples.

In addition, 81.92% (95% CI 75.55–86.93) of the *E. coli* isolates carried the *tet*A gene, followed by 27.68% (95% CI 21.60–34.71) *tet*B and 8.47% (95% CI 5.11–13.60) *tet*C gene. All the isolates (*n* = 177) were tested for the presence of three penicillin resistance genes such as *bla*_SHV_, *bla*_CMY_ and *bla*_TEM_. Among them, 88.14% (95% CI 82.48–92.17) of isolates harboured *bla*_TEM_ gene, followed by *bla*_CMY_ (7.91, 95% CI 4.67–12.93) and the *bla*_SHV_ (5.65%, 95% CI 2.98–10.21) gene. Besides, the prevalence of *aac*(3)‐IV, *sul*1 and *ere* (A) genes was 42.37% (95% CI 35.33–49.74), 72.32% (95% CI 65.29–78.40) and 83.62% (95% CI 77.41–88.39). The ARG profile is presented in Table 4 and Figure 6.

**TABLE 4:** Distribution of antimicrobial resistance genes (ARGs) (*n* = 177) in *Escherichia coli* isolates.

Antimicrobials	Resistance genes	No. of resistant isolates (*n*)	Percentage (95% CI)
Gentamicin resistance	*aac*(3)‐IV	75	42.37 (35.33–49.74)
Sulphonamide resistance	*sul*1	128	72.32 (65.29–78.40)
Penicillin resistance	*bla*_SHV_	10	5.56 (2.98–10.21)
	*bla*_CMY_	14	7.91 (4.67–12.93)
	*bla*_TEM_	156	88.14 (82.48–92.17)
Erythromycin resistance	*ere* (A)	148	83.62 (77.41–88.39)
Tetracycline resistance	*tet* A	145	81.92 (75.55–86.93)
	*tet* B	49	27.68 (21.60–34.71)
	*tet* C	15	8.47 (5.11–13.60)

### 3.4 Distribution of VAGs

**FIGURE 7:**
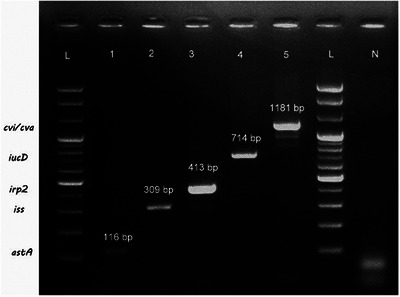
Result of the polymerase chain reaction (PCR) assay for the virulence‐associated genes of *Escherichia coli* isolates tested; Lane L: 1 kb plus DNA ladder; Lane N: negative control; Lane 1–5: samples.

All the *E. coli* isolates (*n* = 177) were tested for the presence of VAGs of APEC. A total of eight virulence genes were tested in this study. Among them, *ast*A genes (also known as EAST1 or heat‐stable cytotoxin associated with enteroaggregative *E. coli* [EAEC]) were carried by 56.5% (95% CI 49.13–63.59) of *E. coli* isolates. *E. coli* genes responsible for aerobactin synthesis, *iucD*, and increasing survival of *E. coli* in the serum, *iss*, were found in 31.07% (95% CI 24.71–38.24) and 21.47% (95% CI 16.03–28.12) of *E. coli* isolates, respectively. Besides, iron‐responsible protein (*irp*2) and colicin V (*cva/cvi)* were found in 15.82% (95% CI 11.13–21.96) and 3.39% (95% CI 1.40–7.36) isolates in this study. *pap*C, *tsh* and *vat* were not detected among *E. coli* isolates. The VAG profile is presented in Table 5 and Figure 7.

**TABLE 5:** Frequency of virulence‐associated genes (VAGs) of *Escherichia coli* isolated from broiler (*n* = 177).

VAGs	Positive (%)	95% CI
*ast*A	100 (56.50)	49.13–63.59
*iss*	38 (21.47)	16.03–28.12
*irp*2	28 (15.82)	11.13–21.96
*pap*C	0	0
*iuc*D	55 (31.07)	24.71–38.24
*tsh*	0	0
*Vat*	0	0
*cva/cvi*	6 (3.39)	1.40–7.36

## 4 DISCUSSION

The comprehensive examination of *E. coli* in broiler cloacal samples conducted in this study sheds light on the complex interplay among poultry health, AMR and virulence factors. The phenotypic characteristics of recovered *E. coli* isolates were similar to a previously reported study by Algammal et al. (2022). In addition, the prevalence of *E. coli* in this study was 81.94%, which is higher than previous studies reported from Bangladesh (Dutta et al., 2020; Hossain et al., 2008; Sarker et al., 2019). The variations in *E. coli* prevalence may be influenced by numerous factors, including geographical location, farm biosecurity practices, isolation methods, farm hygiene and management practices (Ievy et al., 2020).

In this study, all *E. coli* isolates exhibited varying degrees of resistance to important antimicrobials, including ceftriaxone, ampicillin, erythromycin, gentamicin, ciprofloxacin, neomycin, tetracycline and sulfamethoxazole/trimethoprim. *E. coli* isolates exhibited the highest (94%) resistance to ampicillin and the lowest (19%) resistance to ceftriaxone. More than 80% of the isolates showed resistance to tetracycline, erythromycin, ciprofloxacin and sulfamethoxazole/trimethoprim. These resistance patterns align with the previous studies (Al Azad et al., 2019; Das et al., 2021; Subedi et al., 2018; Sarker et al., 2019). However, Al Azad et al. (2019) reported 100% resistance to ampicillin, tetracycline, erythromycin, ciprofloxacin and sulfamethoxazole/trimethoprim. The high resistance level of these antimicrobials in broilers reflects the indiscriminate use of antimicrobials in broiler production by farmers in Bangladesh (Hassan et al., 2021). In contrast, ceftriaxone showed lower resistance (19%), which is expected for this type of cephalosporin as it is not used in broiler farms of Bangladesh (Hasan et al., 2011; Imam et al., 2020).

Furthermore, nine ARGs were screened and their prevalence was similar to a previous study from Bangladesh (Al Azad et al., 2019) but higher than a study reported from Iran (Momtaz et al., 2012). All the *E. coli* isolates in this study were MDR. However, this study could not classify the isolates as extensively drug resistant (XDR) or pan‐drug resistant (PDR) because only a few antimicrobials were tested. According to Magiorakos et al. (2012), an adequate number of antimicrobials must be tested for *Enterobacteriaceae* to classify XDR and PDR.

Regarding the MDR patterns, the highest percentage of isolates were resistant to five or six antimicrobial classes and carried all ARGs tested in this study. It represents the misuse of antimicrobials in the poultry sector of Bangladesh. Resistance can spread through conjugation, transformation and transduction processes, making it possible for highly resistant isolates from broiler farms to transfer resistance markers to other bacteria. Apart from these, resistance is associated with other processes, including growth in the stationary phase, biofilms and persistence (Amer et al., 2018). This transfer occurs to humans through the food chain, mainly by consuming inadequately cooked meat or handling meat unsafely during processing (Nath et al., 2023) and is widely circulated to the environment through poultry waste (Elbehiry et al., 2022). Al Sattar et al. (2023) indicated that increasing farmers’ awareness of antimicrobials has been improved AMR conditions in the farming environment.

In this study, PCR analysis revealed that most of the isolates carried varying amounts of VAGs. The most prevalent VAG identified was *ast*A (56.5%), commonly found in EAEC pathotypes (Sidhu et al., 2013). The genes *iuc*D and *irp*2, related to iron acquisition, were present at frequency of 31.07% and 15.82%, respectively. This aligns with findings from Subedi et al. (2018), reported higher frequency of *iuc*D (97.3%) compared to *irp*2 gene (73.3%) in Nepal. Additionally, the serum survival gene (*iss*) and colicin V plasmid operon gene (*cva*/*cvi*) were detected at frequencies of 21.47% and 3.39%, respectively. In contrast, *pap*C, *tsh* and *vat* were not detected among the *E. coli* isolates.

The overall frequency of VAGs in this study was lower compared to reports from Nepal (Subedi et al., 2018), Jordan (Ibrahim et al., 2019) and Korea (Kwon et al., 2008). Although no isolates in this study were APEC, but most of the isolates carried two to three VAGs. These genes, often chromosomal or plasmid‐mediated and integrated into PAI, cause colibacillosis (De Carli et al., 2015). They contribute to tissue damage, colonization and inflammatory reactions within the host, allowing pathogens to evade host defences, thus escalating the pathogenesis of MDR *E. coli*, thereby limiting treatment options (El‐Baz et al., 2022; Shafiq et al., 2022).

The acquisition of VAGs usually occurs through horizontal gene transfer (Hacker & Kaper, 2000; Ochman et al., 2000), which may explain the absence or the low prevalence of certain VAGs in this study. The presence of virulence markers in *E. coli* is genetically similar with different pathogenic clones, indicating their potential for transferring virulence genes to pathogenic *E. coli* clones in humans and animals (Ahmed et al., 2020). However, several studies have taken an “integrated poultry production” approach and suggested that broiler breeders and hatcheries may be significant reservoirs of early APEC infections via environmental contamination or vertical transmission (Giovanardi et al., 2005; Petersen et al., 2006).

This study highlights the urgency of addressing resistance and virulence in poultry, emphasizing the significance of policy changes, strong legislation, law enforcement and collaborative efforts. By aligning with global standards and research‐driven strategies, the poultry industry can effectively combat AMR, protect poultry health and mitigate potential public health risks.

Further studies on AMR using all listed and available antimicrobials for *E. coli* are recommended to understand the current status of AMR. Additionally, investigations using the one health approach are urgently needed in Bangladesh to identify the spread of AMR pathogens, including those affecting clinical patients. A holistic farm‐to‐fork approach would be valuable for future research to identify contamination points in meat and assess consumer exposure to AMR pathogens by identifying potential hazards along the food chain. Moreover, additional studies on biofilm characteristics should be conducted to assess the potential pathogenicity of the isolates.

## 5 CONCLUSION

This study investigated the occurrence of VAGs in *E. coli* and identified their AMR patterns. The widespread AMR of *E. coli* and the identification of ARGs highlighted the importance of monitoring the spread of ARGs in poultry farms and in the environment. The presence of VAGs in MDR *E. coli* limits the treatment options for pathogenic strains. Routine surveillance, monitoring and stakeholder awareness should be strengthened to prevent the dissemination of MDR *E. coli* as well as minimize possible risks to public health.

## AUTHOR CONTRIBUTIONS

**Md. Sirazul Islam**: Conceptualization; data curation; methodology; project investigation; ncbi data submission; visualization; writing‐ original draft; writing‐review and editing. **Chandan Nath**: Data curation; methodology; formal analysis; visualization; writing‐ review; and editing. **F. M. Yasir Hasib** and **Tahia Ahmed Logno**: Data curation; methodology. **Md. Helal Uddin**: Data curation; formal analysis. **Mohammad Mahmudul Hassan**: Validation; writing‐review and editing. **Sharmin Chowdhury**: Funding acquisition; project administration; supervision; validation; writing‐review and editing.

## CONFLICT OF INTEREST STATEMENT

The authors declare no conflicts of interest.

## ETHICS STATEMENT

Licensed veterinarians carried out all investigations and sample collection procedures from the implementing institute. Before data collection and sampling, farmers were informed about the research and asked to participate willingly. The Chattogram Veterinary and Animal Sciences University's (CVASU) Ethical Committee (EC) reviewed the proposal and gave ethical approval on 18 March 2019, for conducting this research [Memo# CVASU/Dir(R&E) EC/2019/39(2/6/6)]

### PEER REVIEW

The peer review history for this article is available at https://publons.com/publon/10.1002/vms3.70032

## Data Availability

The five virulence‐associated gene (*ast*A, *iss*, *irp*2, *iuc*D and *cvi/cva*C) sequences were submitted to GenBank of the National Center for Biotechnology Information (NCBI) under accession numbers MT928164‐MT928166 and MT982360‐MT982361.
